# Publication ethics in practice

**DOI:** 10.4314/gmj.v55i2.1

**Published:** 2021-06

**Authors:** Joseph Bonney, Peter Donkor

**Affiliations:** Directorate of Emergency Medicine, Komfo Anokye Teaching Hospital, Kumasi; Department of Surgery, Kwame Nkrumah University of Science and Technology, Kumasi

Publication ethics ensure that publications are truthful, due credit is given to contributors, the rights of participants are protected, applicable rules and guidelines are adhered to, and the public's trust is nurtured.

There is usually some confusion between research ethics and publication ethics. The two are distinct and yet linked. Publication of research works is the downstream part of the research. Research that defies the principles of research ethics cannot satisfy the requirements of publication ethics.

Ethical breaches in communicating research findings are increasing in recent times.^1^ Unfortunately, standards in publications may not necessarily be assured by all these journals. With the increase in the numbers of journals, some journals publish articles without verifying the authenticity of the submitted manuscript's ethical clearances.^2^ In recent times, the Ghana Medical Journal has seen examples of authors trivialising the need for ethical approval before conducting research involving human or animal participants. A particular area of concern is the need to present research proposals to the Ethics Review Committees (ERC) or the Institutional Review Board (IRB) before starting their study, even when the institutional research policy states otherwise. The approval or waiver of the ERC or IRB is required before study initiation, not after the report is written.

In terms of human and animal research, ERCs or IRBs provide the mechanism or platform for ensuring the principles underlying research ethics. In doing so, research participants are protected, and institutions protect themselves from potential charges of infringements on the rights of participants. In the same way, researchers should be minded ethically documenting their findings. The Committee on Publication Ethics (COPE) lists examples of publication misconduct as unethical research, data fabrication, falsification or manipulation of data, plagiarism, selective reporting, redundant publication, inappropriate authorship, and undeclared conflicts of interest as examples of research and publication misconduct.^3^ Deviations from these principles will discredit authors and their institutions. Most academic institutions appoint research integrity officers who adjudicate matters relating to research and publication.

A recent example of a breach of research ethics is the publication of unverified data on the COVID-19 pandemic. This led to the concern about hydroxychloroquine and the subsequent withdrawal of articles from reputable journals.^4^

There are many ethical codes on research, but all are based on beneficence, non-maleficence, justice, autonomy. Thus, the problem is not the type of ethical misconduct but rather why people choose to research without proper ethical approvals. Reasons may include the cumbersome processes for submitting to the review committees, especially where multiple hard copies of documents are required.^5^ Other reasons include the cost of ethical approval, lack of knowledge about ethical research principles, trivialising the need for ethical approval in retrospective research and case studies, and the lack of enforcement of rules of ethical clearance.^6^

Previously, it used to be difficult for some journals to detect whether a manuscript had gone through the proper ethical review process. This is quickly changing with advances in technology. An example is a requirement that clinical trials should be registered with the appropriate national or international registry. When a protocol is registered, it becomes evident that the researchers will provide evidence of ethical clearance before the clinical trial can start. Many countries have established bodies for regulating clinical trials. Other countries have established a registry National Health Research Committees^7^ or systems to verify the credentials of research ethics committees. Other institutions have a database on ethically cleared documents where researchers upload a copy of the ethical clearance letter or certificate.

It is the responsibility of academic and research institutions to ensure that research and publications undertaken by their staff meet institutional, national, and international best practices.^8^ In this regard, there should be established Standard Operating Procedures for ERC/IRB that can be verified. Institutions must also establish support offices for the conduct of research and scholarly publications. In addition, Research Integrity Officers have a role in ensuring that research and publications conform to international best practices, and education on research and publication integrity is established in their institutions.

Most journals have a statement about research integrity in their publication policies. There is also a statement about publication ethics and the consequences of misconduct. The authors' responsibility is to read and understand journal policies, usually found in the Instructions to Authors. In addition, it is usual for journals to request that the name of the institutional ethics committee(s), reference of the ethics approval letter(s) and a statement about informed consent of participants be included in the manuscript.

At the institutional level, it will be useful to have guidelines on research ethics indicating which type of research should be reviewed by the ERC/IRB. It is much safer for researchers to seek an ERC/IRB decision before commencing research work.

There should be a global charge to promote research and publication ethics. This will allow researchers to work within a safe space to promote good and safe research and allow for journal publications to withstand scrutiny and avoid the risk of having retractions, which will tarnish the image of the authors, their institutions, and the journal at large.
